# Positive Attitudes to Pediatric HIV Testing: Findings from a Nationally Representative Survey from Zimbabwe

**DOI:** 10.1371/journal.pone.0053213

**Published:** 2012-12-28

**Authors:** Raluca Buzdugan, Constancia Watadzaushe, Jeffrey Dirawo, Oscar Mundida, Lisa Langhaug, Nicola Willis, Karin Hatzold, Getrude Ncube, Owen Mugurungi, Clemens Benedikt, Andrew Copas, Frances M. Cowan

**Affiliations:** 1 University College London, Research Department of Infection & Population Health, London, United Kingdom; 2 University of California, Berkeley, School of Public Health, Berkeley, California, United States of America; 3 University of Zimbabwe, ZAPP-UZ, Community Medicine, Harare, Zimbabwe; 4 National AIDS Council, Harare, Zimbabwe; 5 Africaid, Harare, Zimbabwe; 6 Population Services International, Harare, Zimbabwe; 7 Ministry of Health and Child Welfare, Harare, Zimbabwe; 8 UNFPA, Harare, Zimbabwe; Vanderbilt University, United States of America

## Abstract

**Objective:**

Early HIV testing and diagnosis are paramount for increasing treatment initiation among children, necessary for their survival and improved health. However, uptake of pediatric HIV testing is low in high-prevalence areas. We present data on attitudes towards pediatric testing from a nationally representative survey in Zimbabwe.

**Methods:**

All 18–24 year olds and a proportion of 25–49 year olds living in randomly selected enumeration areas from all ten Zimbabwe provinces were invited to self-complete an anonymous questionnaire on a personal digital assistant, and 16,719 people agreed to participate (75% of eligibles).

**Results:**

Most people think children can benefit from HIV testing (91%), 81% of people who looked after children know how to access testing for their children and 92% would feel happier if their children were tested. Notably, 42% fear that, if tested, children may be discriminated against by some community members and 28% fear their children are HIV positive. People who fear discrimination against children who have tested for HIV are more likely than their counterparts to perceive their community as stigmatizing against HIV positive people (43% vs. 29%). They are also less likely to report positive attitudes to HIV themselves (49% vs. 74%). Only 28% think it is possible for children HIV-infected at birth to live into adolescence without treatment. Approximately 70% of people (irrespective of whether they are themselves parents) think HIV-infected children in their communities can access testing and treatment.

**Conclusions:**

Pediatric HIV testing is the essential gateway to prevention and care services. Our data indicate positive attitudes to testing children, suggesting a conducive environment for increasing uptake of pediatric testing in Zimbabwe. However, there is a need to better understand the barriers to pediatric testing, such as stigma and discrimination, and address the gaps in knowledge regarding HIV/AIDS in children.

## Introduction

An estimated 3.4 million children under the age of 15 were living with HIV globally in 2010 and 90% of them were living in Sub-Saharan Africa [1]. In Zimbabwe, 2.8% (138,642) of children are estimated to be living with HIV [2]. Given that most of these children acquired HIV from their mothers during pregnancy, labor or breastfeeding, and efficacious interventions for the prevention of mother-to-child HIV transmission have had limited coverage and impact, 330,000 children were newly infected with HIV in 2011, most of them living in sub-Saharan Africa [3].

In the absence of diagnosis and treatment, more than half of HIV infected children die by their second birthdate [4]. Initiating antiretroviral therapy (ART) of HIV positive children prior to their first year can reduce mortality by 76% and HIV progression by 75% [5], however only 456,000 children from low- and middle-income countries were receiving ART in 2010, an estimated 23% of the 2.02 million children in need of ART [1].

Early HIV testing and diagnosis are paramount for increasing ART initiation among children, necessary for their survival and improved health. However, uptake of HIV testing among children is low even in high HIV prevalence areas [6]. In Zimbabwe only 14% of infants born to HIV positive mothers were tested for HIV by two months of age [1] and data on uptake of HIV testing among older children is limited. Perception of pediatric HIV testing in terms of potential risks and benefits is an important factor in one’s decision to have their child(ren) tested for HIV infection. For instance, fear of stigma and discrimination have been shown to negatively affect uptake of infant testing and care in Africa [7], [8] and Zimbabwe in particular [9]. Previous studies have documented high acceptance of pediatric testing among Cote d’Ivoire health workers [10] and Zimbabwe adolescents [11] and low acceptance among Kenyan caregivers [12]. However, these have largely been small-scale studies focused on specific subpopulations. This paper presents data regarding knowledge and attitudes towards pediatric HIV testing, as reported during a nationally representative household survey of 18–49 years olds in Zimbabwe.

## Methods

### Survey Methodology

A cross-sectional survey of 18–49 year olds was conducted in all ten provinces in Zimbabwe in July 2011-January 2012, representing the final of three surveys conducted for the evaluation of Zimbabwe’s National Behavioural Change Programme (NBCP). The evaluation of NBCP covered 16 districts from Mashonaland East, Masvingo, Matebeleland North and Midlands provinces (four districts selected per province) surveyed during the baseline survey (three randomly selected urban/peri-urban enumeration areas (EAs) and seven rural EAs per district). This survey was expanded beyond the geographic scope of the original evaluation to include all ten provinces in order to collect information on exposure to mass media messages nationally. (The expanded final survey also covered Manicaland, Mashonaland Central, Mashonaland West and Matebeleland South provinces (three urban/peri-urban EAs and seven rural EAs randomly selected per province), and Harare and Bulawayo cities (30 and 10 randomly selected EAs per city respectively)).

After enumerating all 18–49 year olds living in the selected EAs, all 18–24 year olds (“youth”) and a proportion of 25–49 year olds (“adults”) (approximately 40 per age group per EA) were invited to visit a centrally located survey site to self-complete an anonymous questionnaire on a personal digital assistant using audio-computer assisted self-interviewing software, following written informed consent. Complete questionnaires were collected among 16,719 people (75% of eligibles). The study procedures were approved by the institutional boards of University College London, United Kingdom and the Medical Research Council of Zimbabwe.

### Data Analysis

The data were analyzed using Stata 12 (Stata Corp, College Station, TX, USA) after weighting through post-stratification to the 2002 Census. Specifically, the weights were developed by first calculating the percentage of the sample in each cross-classification of gender, age (youth vs. adults), urban/rural status and province. The corresponding percentages of the total population were derived from the census, and for each cross-classification a weight was calculated as the ratio of the census to sample percentages. However, for the four provinces where districts were initially selected and then EAs within these (see above), the post-stratification was performed at the district (rather than province) level and then participants were additionally weighted so that the proportion of the sample in these four provinces matched the population proportion derived from the census. The paper presents results of frequency distributions, cross-tabulations and tests of significance based on the survey functions in Stata.

We briefly explain how we derived some of the variables employed in the analysis. An index measuring HIV knowledge ranging from 0 to 6 was created by adding the answers (1 = yes, 0 = no/don’t know) to six questions: 1) If you look carefully, you can know if someone has HIV (reverse coded); 2) Using condoms can prevent you from being infected by HIV; 3) A person who looks strong and healthy can have HIV; 4) A mother can transmit HIV through breastfeeding; 5) You can get HIV if you share utensils with someone who is infected (reverse coded); and 6) If a mosquito bites you it can infect you with HIV (reverse coded). The index was categorized into low (0–2), medium (3–4), and high knowledge (5–6).

Attitudes for HIV (specifically, expressed HIV-related stigma) were measured using an index computed by adding answers (1 = strongly agree/agree, 0 = strongly disagree/disagree) to 12 questions: 1) I would buy food from an HIV-positive shopkeeper; 2) HIV-positive teachers who are not ill should not be allowed to teach in school (reverse coded); 3) HIV-positive health workers should not be allowed to treat patients (reverse coded); 4) If a family member would become HIV-positive I would want it a secret (reverse coded); 5) HIV/AIDS is the result of sinning (reverse coded); 6) It is a waste of money to train/educate someone with HIV (reverse coded); 7) One would be foolish to marry someone with HIV (reverse coded); 8) People with HIV/AIDS should not be ashamed; 9) Health workers should treat people with AIDS as people with other illnesses; 10) People with HIV should be allowed to participated in social events; 11) It is reasonable for an employer to fire someone with AIDS (reverse coded); and 12) People with HIV/AIDS do not deserve any support (reverse coded). The index was categorized into mostly negative (0–4), medium (5–8), and mostly positive attitudes (9–12).

Perceived HIV-related stigma (1 = yes, 0 = no) in the community was coded ‘yes’ if the participant agreed with at least one of the following two statements: 1) Most people in this community would not buy vegetables from a shopkeeper or food seller if they knew that person had HIV; and 2) Most people in this community would want to remove the teacher in the school if they knew that this person had HIV though they were not sick.

Perceived community norms regarding HIV testing was assessed by adding the answers (1 = strongly agree/agree, 0 = strongly disagree/disagree) to 5 questions: 1) Most people in this community who want to get tested for HIV do not want other people to find out if they get tested (reverse coded); 2) Most people in this community who want to get tested for HIV will tell their spouses/partners that they want to get tested; 3) Most people in this community want to get tested for HIV; 4) Most people in this community get tested for HIV only if they are sick (reverse coded); and 5) In this community people think that young couples should go for an HIV test before getting married. The index was categorized into mostly negative (0–1), medium (2–3), and mostly positive perception scores (4–5).

## Results

### Awareness of Pediatric HIV/AIDS

Approximately 45% of people reported that they know children infected with HIV and 40% know children on ART (Table 1). One third of people know children who have been tested for HIV. With respect to perceived access to pediatric HIV testing and care, 68% of people think that HIV infected children in their communities can access testing and 70% that they can access ART.

**Table 1:** Pediatric HIV/AIDS indicators, Zimbabwe.

	Total(n = 17038)
*Awareness of pediatric HIV/AIDS*	
Know HIV positive children	44.3
Know HIV positive children on ART	39.5
Know children who have tested for HIV	33.0
*Perceived access to HIV testing & care*	
Think HIV positive children in their community can access HIV testing	
Yes	68.4
No	11.8
Don’t know	19.8
Think HIV+ children in their community can access ART	
Yes	70.0
No	12.0
Don’t know	18.1
*Knowledge about survival of HIV infected children*	
Think it is possible for some children infected with HIV at birth to live into adolescence without treatment	27.6
If yes, personally know such children	(n = 4691)
	57.9
*Attitudes towards pediatric HIV testing*	
Think that children can benefit from HIV testing	91.2
Afraid that if children are tested for HIV they may be discriminated against by some members of their community	41.9
Ever talked to their children about HIV testing	
Yes	39.6
No	40.6
Don’t look after any children	19.8
*Only those who look after children*	(n = 13631)
Know how to access HIV testing for their children	80.8
Would feel happier if their children were tested for HIV	92.2
Fear that if their children were tested for HIV, they would be found positive	28.3

ART = antiretroviral treatment.

### Knowledge about Survival of HIV Infected Children

While the majority of people surveyed report being aware of HIV/AIDS among children and think that they can access testing and treatment, only 28% think it is possible for children HIV infected at birth to live into adolescence without treatment. Of these, 58% personally know such children.

### Attitudes Towards Pediatric HIV Testing

Most people think that children can benefit from being tested for HIV (91%). Moreover, 81% of parents/guardians know how to access HIV testing for their children and 92% would feel happier if their children were tested. However, only half of people who looked after children talked to them about HIV testing, although in many cases their children may have been too young for such discussions. Notably, 42% fear that, if tested, children may be discriminated against by some community members and 28% of parents/guardians fear that their children are HIV positive.

### HIV Knowledge by Awareness of Pediatric HIV/AIDS

There appears to be an association between awareness of pediatric HIV/AIDS and knowledge about HIV and mother-to-child HIV transmission (Table 2). For example, 45% of people who personally know children on ART reported high knowledge about HIV compared to 35% of those who do not know children on ART. Similarly, 90% of people who know children on ART are aware that mothers can transmit HIV though breastfeeding compared to 79% of people who do not know children on ART. There is strong association between awareness of pediatric HIV and awareness of HIV in their community (where “HIV in the community” is generally synonymous with “adult HIV in the community”, according to anecdotal evidence). Specifically, 90% of people who know HIV positive children also reported that they know someone with HIV, compared to 51% of people who do not know HIV positive children (Table 2). Table 3 indicates that people with better general knowledge about HIV/AIDS and mother-to-child HIV transmission are slightly more likely to know that some children infected with HIV at birth can live into adolescence without treatment, compared to those without such knowledge.

**Table 2:** HIV knowledge by awareness of pediatric HIV/AIDS.

	Know HIV positive children		Know HIV positive children on ART		Know children who tested for HIV	
	No	Yes	No	Yes	No	Yes
	(n = 9449)	(n = 7515)	(n = 10272)	(n = 6718)	(n = 11388)	(n = 5605)
HIV knowledge	p<0.001		p<0.001		p = 0.002	
Low (0–2)	13.8	5.1	13.6	4.3	10.8	8.2
Medium (3–4)	51.1	51.0	51.0	51.1	50.9	51.2
High (5–6)	35.1	44.0	35.4	44.7	38.3	40.5
A mother can transmit HIV through breastfeeding						
	p<0.001		p<0.001		p<0.001	
Yes	79.1	89.2	79.3	90.1	82.4	86.1
No	9.9	6.4	9.9	6.0	8.5	8.2
Don’t know	10.9	4.3	10.7	3.9	9.1	5.7
An HIV positive mother can do something to prevent HIV infection of baby						
	p<0.001		p<0.001		p<0.001	
Yes	76.1	89.9	76.8	90.5	80.2	86.3
No	9.0	4.3	8.9	4.0	7.4	6.0
Don’t know	14.8	5.8	14.3	5.5	12.4	7.6
Know someone who is HIV positive						
	p<0.001					
Yes	50.7	90.4				
No	49.3	9.6				
Know someone on ART			p<0.001			
Yes			48.2	90.2		
No			51.8	9.8		

ART = antiretroviral treatment.

**Table 3:** Knowledge about survival of HIV infected children by knowledge about HIV/AIDS.

		Think it is possible for children HIV infected at birth to live into adolescence without treatment	
		Yes	p value
HIV knowledge			0.010
Low (0–2)	(n = 1685)	24.3	
Medium (3–4)	(n = 8648)	27.2	
High (5–6)	(n = 6614)	29.0	
A mother can transmit HIV through breastfeeding			0.024
Yes	(n = 14206)	27.9	
No	(n = 1422)	28.5	
Don’t know	(n = 1363)	23.3	
An HIV positive mother can do something to prevent HIV infection of baby			<0.001
Yes	(n = 13969)	28.7	
No	(n = 1184)	22.7	
Don’t know	(n = 1842)	22.2	

**Figure 1:**
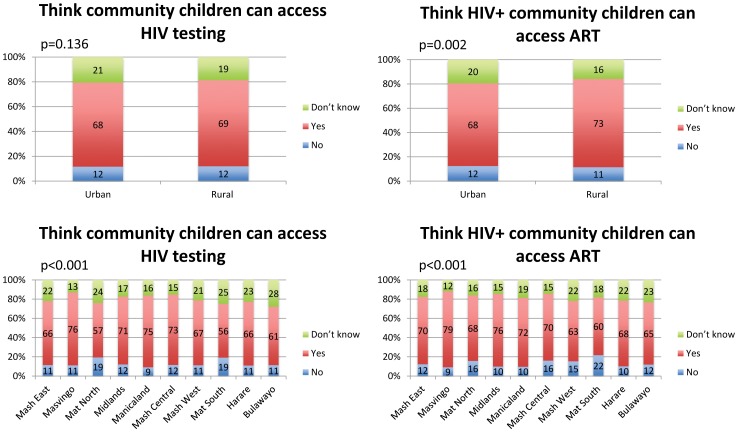
Perceived access to pediatric HIV testing and care by province and rural/ urban status.

Figure 1 indicates substantial variation in the perceived access to pediatric HIV testing and care by province, but not by rural vs. urban status. For instance, 75% of people in Manicaland think parents can access HIV testing for their children compared to 56% in Matebeleland South. However, virtually the same percentage of people share this belief in urban (68%) and rural areas (69%). Similarly, 79% of people in Masvingo vs. 60% in Matebeleland South think parents can access ART for their children.

### HIV-related Stigma by Attitudes about Pediatric HIV Testing

People who think pediatric HIV testing is beneficial are more likely to have tested for HIV themselves (64% vs. 41%). Similarly, 68% of those who know how to access HIV testing for their children had tested for HIV compared to 54% of those who do not know (Table 4). Notably, people who fear discrimination against children who have been tested for HIV are more likely than their counterparts to report HIV-related stigma in their communities (43% vs. 29%) and less likely to report positive HIV testing-related norms (11% vs. 19%). They are also less likely to report positive attitudes to HIV themselves (49% vs. 74%).

**Table 4:** Reported HIV testing and HIV-related stigma in the community by attitudes about pediatric HIV testing.

	Think that children can benefit fromHIV testing			Afraid that if tested for HIV children may be discriminated against		Know how to access HIV testing fortheir children	
	No		Yes	No	Yes	No	Yes
	(n = 1491)		(n = 15472)	(n = 9866)	(n = 7122)	(n = 2611)	(n = 11014)
Perceived HIV-related stigma in the community							
				p<0.001			
Yes				29.3	42.7		
No				70.7	57.4		
Perceived community norms regarding HIV testing							
				p<0.001			
0–1 (Mostly negative)				25.7	28.3		
2–3				55.4	60.7		
4–5 (Mostly positive)				18.9	11.1		
Attitudes to HIV							
				p<0.001			
0–4 (Mostly negative)				3.7	13.8		
5–8				22.6	37.0		
9–12 (Mostly positive)				73.7	49.3		
Ever been tested for HIV							
	p<0.001					p<0.001	
Yes	41.0	63.5				54.1	68.2
No	59.0	36.5				45.9	31.8

### Fear about Pediatric HIV Testing by Own HIV Testing Behaviour

Table 5 examines associations between participants’ reported fear that, if tested, their children would be found HIV positive and their self-perceived HIV risk and reported HIV status. There is only a weak association between fear that their children are HIV-infected and self-perceived HIV risk. People who have tested for HIV themselves are less likely to express fear that their children are HIV-infected (25% vs. 35% of those who have never tested for HIV themselves). Among those who tested for HIV, people who reported to be HIV positive are more likely to fear that they children are HIV-infected (33%) than those who reported being HIV negative (22%); 38% of those who tested for HIV but did not report their status fear that their children are HIV positive.

**Table 5:** Fear about pediatric HIV testing by self-perceived HIV risk and HIV testing.

		Fear that if tested for HIV their children would be found positive	
		Yes	p value
Self-perceived HIV risk			0.004
No risk	(n = 3040)	26.8	
Small or moderate	(n = 5545)	27.8	
Great	(n = 2749)	32.6	
Already know status	(n = 2291)	26.3	
Ever been tested for HIV			<0.001
No	(n = 4694)	34.6	
Yes	(n = 8930)	25.0	
Reported HIV status			<0.001
Negative	(n = 7018)	22.1	
Positive1	(n = 1227)	33.4	
Not reported	(n = 314)	38.2	

The associations discussed in this section were also observed for young men, young women, adult men and adult women separately (data not shown).

## Discussion

We present data from a nationally representative survey from Zimbabwe that indicate positive attitudes towards HIV testing of children; most people think children can benefit from testing and would feel happier if their children (or the children in their care) had been tested. These findings, coupled with the fact that four fifths of people who look after children know where to access testing for their children, suggest a conducive environment for increasing uptake of pediatric testing. To the best of our knowledge, this is the first paper to present national-level data on attitudes to pediatric HIV testing from a high prevalence country.

High acceptance of pediatric testing was documented among health care workers from Cote d’Ivoire [10]. A study that examined actual uptake of pediatric testing, albeit in a much smaller sample, found that the majority of adolescents attending acute primary care services and offered provider initiated testing and counseling (PITC) in Harare, Zimbabwe accepted to be tested for HIV [11]. In contrast, almost half of caregivers from Western Kenya refused to have their high-risk children tested for HIV [12].

These data do suggest some potential barriers to pediatric testing, such as parents/guardians’ fear of discrimination as a result of testing (42%) and fear that their children (or the children in their care) are HIV positive (28%). Notably, people who expressed fear that children tested for HIV would be discriminated against were less likely to have positive attitudes to HIV themselves, suggesting that for some people perceived stigma against children may be a “projection” of their own attitudes. Fear of discrimination has been shown to negatively affect uptake to HIV infant testing and care in the region [7], [8], among other factors [13]. The decision to test one’s child is clearly linked to parents’ status and perceived risk, which complicates the decision-making process; people may not only fear discrimination against their tested children (or the children in their care), but also against themselves [9], [14], [15]. A study conducted in primary schools in Harare, Zimbabwe showed that biological parents feared their children’s HIV test could disclose their own HIV status, while guardians did not share these concerns [9].

More than a quarter of people expressed fear that their children (or the children in their care) may be HIV seropositive, which is unlikely given that only 2.8% of Zimbabwean children are estimated to be HIV infected [2]. Therefore, most of these parents/guardians likely have an unfounded anxiety about the status of their children (or the children in their care). This fear is not associated with their perception of their own risk for HIV. People who have tested for HIV themselves and those who report being HIV-negative are less likely to fear that their children (or the children in their care) may be HIV-infected. These findings prompt additional questions beyond the available data, such as: Do parents think their children may be positive because they know the mother did not test for HIV or access care during her pregnancy? To what extent is perception of children’s risk a reflection of their partners’ perceived risk? A qualitative exploration of these fears and their underpinnings is needed to better understand why so many parents fear their children may be HIV-infected. At the same time, these figures may simply be a measure of people’s fear of HIV rather than an indicator of their assessment of the status of their children (or the children in their care).

We document high perceived access to pediatric HIV testing and care. According to our data, perceived access to these services varies by province but not by urban/rural status, which may suggest regional variation in the availability of services. However, data from the Ministry of Health and Child Welfare (MOHCW) suggest that HIV testing and care services are widely available in Zimbabwe in both rural and urban settings and are free of charge. Specifically, the more efficacious regimen for the prevention of mother-to-child HIV transmission (PMTCT) recommended by the World Health Organization (which emphasizes the need for early infant diagnosis and children’s enrollment into care) has been rolled out throughout the country since mid-2011. In addition, HIV testing for children over 18 months as well as adults is available at all health facilities in Zimbabwe, although for children under 16 years testing requires parental or guardian consent.

Over a quarter of people are aware that children infected with HIV at birth can live into adolescence without treatment, and almost 60% of them personally know such children, indicating the need for interventions to incorporate information about the chances of survival of HIV infected children in their materials and messaging. Although long-term survival following mother-to-child HIV transmission was underestimated during the early HIV epidemic, about a quarter of infected infants has been shown to live 10 years or more without ART [16]. Given high HIV prevalence in the absence of PMTCT interventions during the 1990s in many African countries, a substantial minority of children and adolescents are likely HIV positive [17]. Most long-term survivors of mother-to-child HIV transmission remain undiagnosed [16], partly because people are not aware that children can survive untreated mother-to-child HIV transmission [11]. Routine provider-initiated testing among hospitalized children is being advocated given that HIV is the most common cause of adolescent hospitalization in high prevalence countries [18]–[22].

Data indicates associations between awareness and knowledge about pediatric testing, and knowledge about HIV/AIDS in general. Moreover, positive attitudes about pediatric testing seem to be associated with parents/guardians’ testing status. These data suggest that interventions aiming to increase HIV/AIDS knowledge and uptake of testing among adults may have a trickle-down effect on pediatric testing in the form of positive attitudes.

Reported findings are based on nationally representative data collected in all ten Zimbabwe provinces. However, weighting the data using 2002 Census indicators presents some limitations, given the high migration within Zimbabwe since 2002. While the examination of the above-mentioned associations disaggregated by gender and age groups indicated similar patterns, other underlying factors may explain some of the associations observed. All questions about pediatric testing asked about “children” generally, allowing a variation in participants’ interpretation of the children’s age range (e.g. including or excluding infants and/or older adolescents). We are unable to distinguish between the perceptions of biological parents and guardians or to take into account the age or HIV status of participants’ children (or the children in their care), as these data were not collected as part of the survey.

Pediatric HIV testing is the essential gateway to prevention and care services for children. Our data indicate positive attitudes to testing children in Zimbabwe. However, positive attitudes to medical procedures do not always concretize as high uptake (as is the case for male circumcision as an HIV preventive method). Hence, there is a need to better understand the barriers to pediatric testing, such as stigma and discrimination, and address the gaps in knowledge regarding HIV/AIDS in children.
